# Molecular Dynamics Study on the Sintering Mechanism and Tensile Properties of Novel Cu Nanoparticle/Graphene Nanoplatelet Composite Solder Paste

**DOI:** 10.3390/ma17194759

**Published:** 2024-09-27

**Authors:** Xuezhi Zhang, Jian Gao, Lanyu Zhang, Yun Chen, Yu Zhang, Kai Zhang

**Affiliations:** State Key Laboratory of Precision Electronic Manufacturing Technology and Equipment, School of Electromechanical Engineering, Guangdong University of Technology, Guangzhou 510006, China

**Keywords:** Cu nanoparticle, graphene nanoplatelet, molecular dynamics, mechanical properties, deformation, strengthening mechanism

## Abstract

The sintering process of Cu nanoparticle (Cu NP)/graphene nanoplatelet (GNP) composite solder paste was thoroughly investigated in this work through molecular dynamics simulations. The tensile properties of the sintered Cu NP/GNP composite solder paste were considered by using the uniaxial quasi-static tensile simulation method. The impact of sintering temperature, strain rate, and GNP addition on the tensile properties of Cu NP/GNP sintered structures was thoroughly investigated. The lattice structure, dislocation evolution, and atomic diffusion of the molecular dynamics results were analyzed using the common neighbor analysis (CNA), dislocation extraction algorithm (DXA), and mean square displacement (MSD) methods. The results of the post-processing analysis showed that the addition of GNP and the sintering temperature have an important influence on the mechanical properties of Cu NP/GNP sintered structures. In addition, the incorporation of GNP can significantly improve the mechanical properties of sintered Cu NP/GNP composite solder paste. More specifically, the tensile strength and fracture strain of the sintered composite solder paste will be increased by increasing the tensile strain rate. The strengthening mechanism of the sintered Cu NP/GNP composite solder paste can be attributed to the dislocation strengthening mechanism. Our study provides valuable insight for the development of high-performance composite solder paste with enhanced mechanical properties.

## 1. Introduction

Solder paste is an interconnect material used in integrated circuit packaging to provide electrical, thermal, and mechanical connections between chips and substrates [1,2]. The first widely used solder paste was lead–tin solder paste, but later, this material was gradually phased out due to the environmental pollution caused by metallic lead [3,4]. Due to the size effect of nanomaterials, silver nanoparticle solder pastes can be sintered at relatively low temperatures and still meet the packaging requirements for good mechanical, thermal, and electrical properties. Therefore, silver nanoparticle solder pastes have been increasingly used in IC packaging [5,6]. However, due to the fact that the cost of metallic silver materials is much higher than that of copper materials, copper nanoparticle solder paste is gradually being considered a substitute for silver nanoparticle solder paste to meet the industry’s requirements for the low-cost control of products [7,8,9]. Copper nanoparticle solder paste is mainly a paste-like material that is composed of organic solvents such as copper nanoparticles (Cu NPs) and terpineol. To achieve higher mechanical, thermal, and electrical properties, various reinforcing materials, such as graphene and silicon carbide, have been added to pure copper nanoparticle solder paste to obtain a new type of composite solder paste with higher performance [10,11,12].

By now, due to its excellent physical and chemical properties, graphene material has been widely used in various fields [13,14,15]. Various works in the literature, including those on graphene/polymer composites and graphene/metal composites, have extensively explored the excellent properties of graphene [16,17,18]. Zhang et al. [19] improved the thermal conductivity of a sintered body by adding graphene to copper nanoparticle solder paste, and concluded that the C-O-Cu bond formed at the interface between graphene and Cu NPs is crucial for improving the thermal, electrical, and mechanical properties of the thermal interface material. In another interesting work, Yong et al. [20] doubled the electromigration life of solder joints by adding 0.2 wt% graphite oxide (GO) to SnAgCu lead-free solder paste. Ke et al. [21] prepared graphene nanosheet/Cu composites by using the vacuum filtration and spark plasma sintering methods, and when the volume fraction of the graphene nanosheets reached 35 vol%, the thermal conductivity of the composite material was enhanced to 525 W/mK, which is 50% higher than that of the Cu matrix. The feasibility of adding graphene has been elucidated to improve the mechanical, electrical, and thermal properties of composite solder paste. However, as far as the sintering of nanocomposite solder paste is concerned, most of the employed sintering experiments are performed based on macroscopic analysis without considering microscopic molecular level analysis. The dynamic diffusion evolution during the sintering process can be observed through molecular dynamics, which is very important to reveal the sintering mechanism and the reinforcement mechanism of the graphene on the nanocomposite solder paste.

Molecular dynamics is a computational simulation method based on Newtonian mechanics that integrates several disciplines such as physics and mathematics. Currently, it is widely used to study the microscopic diffusion mechanism of nanoparticles during the implementation of the sintering process [22,23,24,25]. Liu et al. [26] performed molecular dynamics simulations on a low-temperature sintering model containing two substrates and several Cu NPs. By comparing the microstructure and dislocation distribution of the 3 nm, 4 nm, and 5 nm models, the influence of the copper particle size on the sintering process and agglomeration mechanism was investigated. Gu et al. [27] discussed the melting behavior of a single nanoparticle, and analyzed the structural evolution and the morphological changes in nanoparticles with the same and different sizes during the sintering process. The authors proved that the sintering process of the differently sized nanoparticles benefited from the wetting behavior of the small-sized nanoparticles on the surface of the large-sized nanoparticles. Jiang et al. [28] investigated the structural evolution and sintering mechanism of aluminum nanoparticles by carrying out molecular dynamics simulation. Yang et al. [29] sintered Cu NPs with sizes of 4, 5, and 6 nm at temperatures of 300, 500, and 700 K and investigated the impact of particle size and sintering temperature on the aggregation of the nanoparticle system. Particularly, simultaneous uniaxial tensile/compression and shear simulations at a constant strain rate were conducted to obtain the mechanical properties of the model, such as Young’s modulus and yield strength. The feasibility of using molecular dynamics-based methods to study the micro-sintering mechanism of nanoparticles and the mechanical properties of sintered bodies has also been demonstrated in previous studies [30,31]. However, the sintering mechanism and the reinforcement mechanism of the graphene on the nanocomposite solder paste is still unclear. Therefore, unlike the current research on graphene–nanocopper composite solder paste, which is mainly conducted using macroscopic experiments on the new material, this paper conducted a microscopic study on the new material using the molecular dynamics method to further elucidate the microscopic mechanism of the sintering of graphene-composite copper nanoparticle solder paste. Through the molecular dynamics simulations performed, we can elucidate the diffusion mechanism of the sintering process of the composite solder paste and the tensile mechanical properties of the sintered body.

In this paper, a molecular dynamic sintering model for Cu NP/GNP composite solder paste was established using the molecular dynamics method, and the sintering mechanism of the Cu NP/GNP composite solder paste was thoroughly investigated. By using the common neighbor analysis (CNA) tool and the dislocation extraction algorithm (DXA) analysis tool, the crystal structure and dislocation evolution during the implementation of the sintering process were also examined. The atomic diffusion activity during the sintering process was then studied by analyzing the corresponding atomic mean square displacement (MSD) plots. Finally, uniaxial tensile tests were performed on the sintered body of the composite solder paste to determine its tensile mechanical properties. By comparing the tensile tests of the composite solder paste and the sintered body of the pure copper nanoparticle solder paste, the underlying strengthening mechanism of the graphene on the composite solder paste was revealed. On top of that, considering the different sintering temperatures and tensile strain rates, the impact of the different sintering and tensile parameters on the tensile mechanical characteristics of the sintered bodies was explored.

## 2. Molecular Dynamics Modeling and Simulations

### 2.1. Molecular Dynamics Model

**Figure 1:**
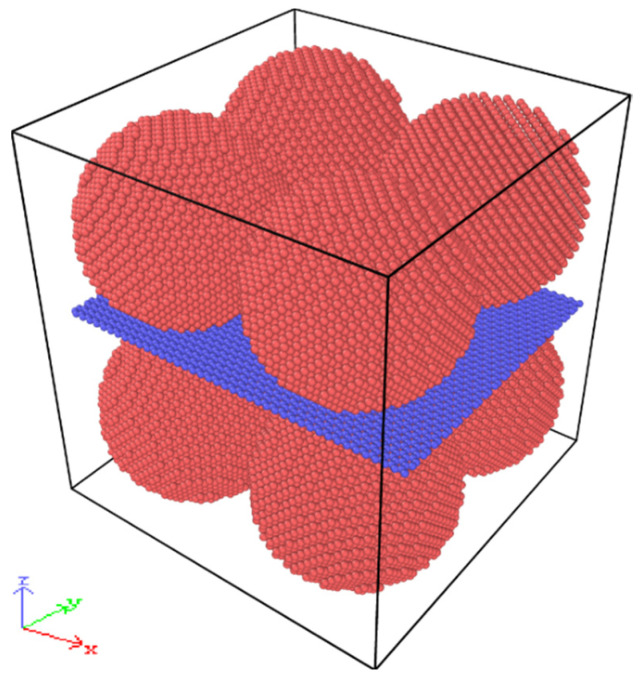
Initial model of the Cu NP/GNP composite paste.

The Large-scale Atomic/Molecular Massively Parallel Simulator (LAMMPS, developed by Sandia National Laboratories, Albuquerque, NM, USA) is widely used by the scientific community to carry out molecular dynamics simulations. Post-processing of the atomistic data obtained from molecular dynamics simulation was conducted using the Open Visualization (OVITO V2.9.0) software. Since the actual sintering model is very complicated, the simulation system was simplified appropriately for the convenience of calculation. The simulation system consists of eight spherical Cu NPs with a GNP embedded in the cubic cell, as can be observed in Figure 1. The initial size and number of atoms of the model are listed in Table 1. The distance between the Cu NPs was set to 2 Å to prevent atoms from overlapping, and the Cu NPs and GNP were separated by a distance of 3 Å.

Three different potential functions were used to describe the interaction between the various atoms in the composite solder paste. The embedded atom method (EAM) potential was also employed for the description of the interaction of copper atoms. Based on the literature [32,33], the potential can be estimated as follows:

(1) $$E^{EAM}=\sum_{i}\left[\frac{1}{2}\sum_{j\neq i}\varphi \left(r_{ij}\right)+F\left({\bar{\rho }}_{i}\right)\right]$$

(2) $${\bar{\rho }}_{i}=\sum_{j\neq i}\rho \left(r_{ij}\right)$$

where ***φ*** represents the pair potential between two atoms, ***i*** and ***j***, with a separation distance of $r_{ij}$, ***F*** denotes the embedding energy, and ***ρ*** stands for the electron cloud density function.

The adaptive intermolecular reactive empirical bond order (AIREBO) potential was employed to describe the interaction between carbon atoms in graphene nanoplatelets. It can be expressed as follows [34]:

(3) $$E^{AIREBO}=\frac{1}{2}\sum_{i}\sum_{j\neq i}\left[E_{ij}^{REBO}+E_{ij}^{LJ}+\sum_{k\neq i,j}\sum_{l\neq i,j,k}E_{kijl}^{TORSION}\right]$$

where the E^AIREBO^ potential is made up of three terms. The E^REBO^ term in the E^AIREBO^ potential endows reactive capabilities upon the model and describes only short-ranged C-C interactions (r < 2Å). Furthermore, the E^LJ^ term uses a form similar to the standard Lennard-Jones potential [35] to give longer-ranged interactions (2Å < r < cutoff), whereas the E^TORSION^ term is an explicit 4-body potential describing different dihedral angle preferences in hydrocarbon configurations.

The interaction between the C and Cu atoms can be described by the Lennard-Jones (L–J) potential function [35] using the following function:

(4) $$E^{LJ}=4\varepsilon \left[{\left(\frac{\sigma }{r_{ij}}\right)}^{12}+{\left(\frac{\sigma }{r_{ij}}\right)}^{6}\right],\;\;\left(r_{ij}<r_{c}\right)$$

where the L–J potential E^LJ^ consists of two parts with opposite effects: the first term (12th power term) represents repulsive energy, and the second term (6th power term) stands for attractive energy. ***ε*** is the depth of the potential well and ***σ*** states the distance between the atoms when the potential is equal to 0. The L-J potential parameters in this work are listed as follows: ***ε*** = 0.02578 eV, ***σ*** = 0.30825 nm, and a cutoff distance of $r_{c}$ = 2.5 ***σ*** [36].

### 2.2. Simulation Details

In this work, the iteration time step in the simulation program was set to 1 fs, and the unit in the simulation program was set to the metal system. In the metal system unit, the temperature, pressure, and energy units were K, bar, and eV, respectively. In the x, y and z directions, the periodic boundary conditions were set.

**Figure 2:**
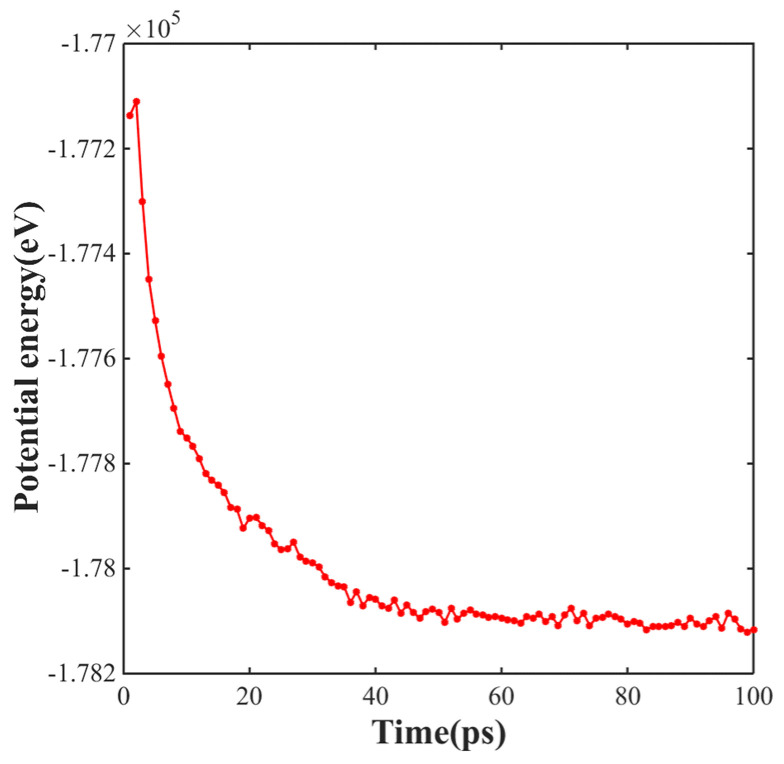
Distribution of the variation curve of the total potential energy for the Cu NP/GNP composite paste model.

Prior to sintering, the molecular dynamics simulation model was relaxed for 50 ps at a temperature of 300 K to reach the equilibrium state of the simulation system. The total potential energy gradually converged during the relaxation process. When the value of the total potential energy tended to be stable, it could be considered that the whole system had basically reached the equilibrium state. Figure 2 shows the variation curve of the total potential energy for the Cu NP/GNP composite paste model, which changed with time during the relaxation process. From the extracted results, it can be seen that the system can remain in an equilibrium state after 50 ps.

The molecular dynamics simulation system uses the Nose–Hoover method for temperature control. After maintaining the target temperature (500, 550, 600, and 650 K), the temperature of the simulation system was gradually lowered to 300 K. In this work, three different strain rates were considered to uniaxially stretch the sintered structures along with the *X*-axis direction, and the strain rates were set to 0.01/ps, 0.005/ps, 0.002/ps, and 0.001/ps, respectively. Taking the model of a 500 K sintering temperature and a 0.01/ps strain rate as an example, the parameter details of the sintering and stretching process are presented in Table 2.

Uniaxial quasi-static tensile simulations were performed on the composite paste after sintering to determine the mechanical properties of the sintered composite paste. The true strain rate of the sintered model during tension was set to 2 × 10^12^/s. During the sintering and deformation simulation, the information for atoms such as atomic coordinates and velocity was dumped every 500 timesteps. The simulation of the system was performed under an NPT-balanced ensemble.

In this work, the crystal structure and dislocation evolution of the model were assessed by using the CNA tool and the DXA analysis method. The atomic diffusion activity was investigated by analyzing the MSD.

## 3. Results and Discussion

### 3.1. Sintering and Strengthening Mechanism

#### 3.1.1. CNA Method

The CNA tool was used to determine the type of atomic structure from the angle of atoms and their surrounding pairs. Through the HA bond type index method, the analyzed atoms can be divided into five categories: Body-Centered Cubic (BCC), Face-Centered Cubic (FCC), Hexagonal Close-Packed (HCP), Cubic Close-Packed (ICO), and OTHER. In the FCC-type crystals, the atomic structure of stacking faults and twin layers is generally the HCP type. The appearance of the HCP lattice structure means the formation of dislocation. Therefore, the defect distribution and dislocation expansion of the model can be observed by CNA.

**Figure 3:**
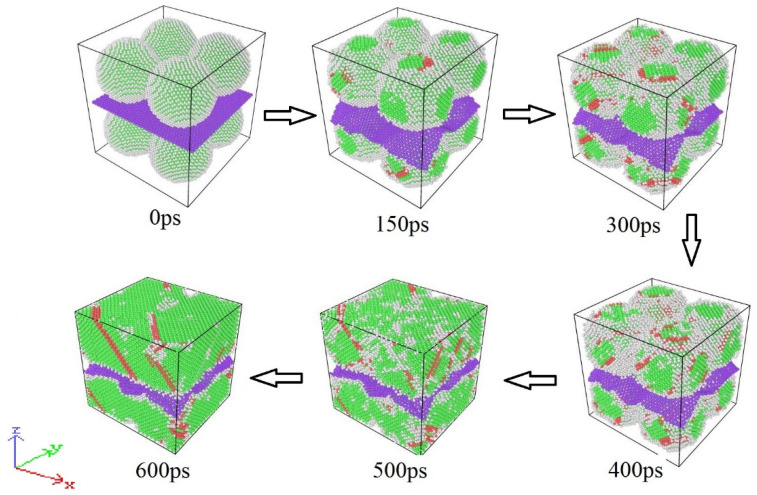
The evolution of the lattice structure during sintering of the composite solder paste.

The atomic configuration of the nanoparticle system was observed at different times to further investigate the sintering mechanism of the nanoparticle composite system. Figure 3 depicts the atomic arrangement of the Cu NP/GNP composite solder paste system sintered at 600 K. The data generated by the simulation were visualized and analyzed using the OVITO (V2.9.0) software package. In Figure 3, the CNA approach was utilized to determine the different crystal structures. The colors green, red, and white indicate the FCC, HCP, and other structures, respectively. As can be seen, at the initial stage of the sintering process, the atoms in the neighboring nanoparticles rapidly converged toward the interface, thus forming an initial sintering neck. Strong attraction between the nanoparticles also took place. Therefore, when nanoparticles make contact with each other, a certain width of the sintering neck will quickly be formed. A Van der Waals force and a metal binding force are part of this attraction. The nanoparticles partially melted off the surface due to the increase in temperature and pressure. This process developed rapidly, hence forming a large number of stacking defects near the sintering neck. As a result, more HCP structures were gradually formed, and the nanoparticle composite system gradually contracted to form a cube shape after 500 ps.

#### 3.1.2. MSD Atomic Diffusion

The sintering mechanism is affected by the applied sintering temperature because the diffusion modes, such as atomic surface diffusion, volume diffusion, and grain boundary diffusion, are activated by increasing the temperature [29]. By taking into account that the slope of the MSD with time is proportional to the diffusion coefficient of the diffusing atoms, the atomic diffusion activity can be studied by analyzing the corresponding atomic MSD plot. To investigate the influence of the sintering temperature on the sintering mechanism, the sintering process was analyzed by MSD at temperatures of 500 K, 550 K, 600 K, and 650 K.

**Figure 4:**
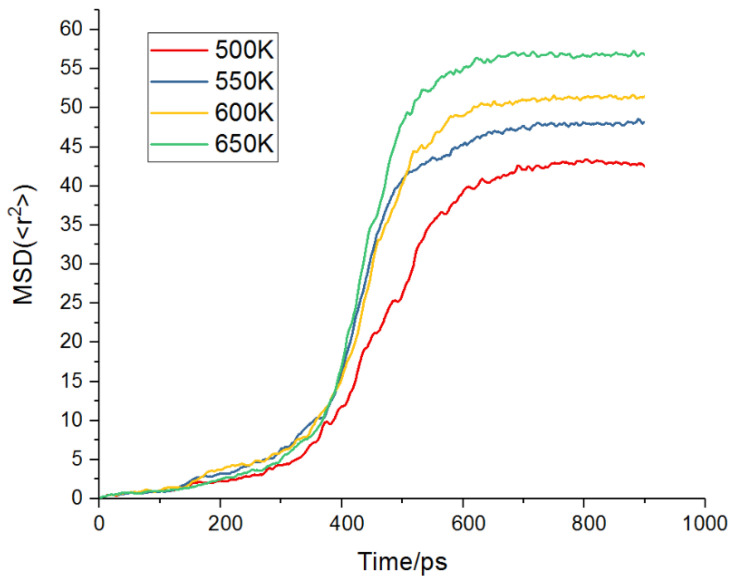
The distribution of the different sintering temperatures (500 K, 550 K, 600 K, and 650 K) on the MSD results of the sintering model.

As shown in Figure 4, atomic diffusion was not significantly activated during the temperature rise and pressure rise of 0–300 ps. However, during the process of 300–600 ps insulation and pressure maintenance, the slope of the MSD diagram was significantly increased, especially in the second half of insulation and pressure maintenance (400–600 ps), which means that the atomic diffusion is mainly concentrated in the insulation and pressure maintenance stage. In addition, the degree of atomic diffusion increased with an increase in temperature. Therefore, compared with the process of maintaining temperature and pressure, the impact of the heating and cooling rates on atom diffusion is not obvious. To improve atom diffusion, the duration of holding the temperature and pressure and the sintering temperature can be appropriately increased to reduce the time taken for the temperature and pressure to rise and the temperature to drop.

#### 3.1.3. Dislocation Extraction Algorithm

DXA can identify all dislocations in the crystal and output dislocation lines. DXA can reveal the dislocation layer in the form of surrounding lines, and can clearly show the dislocation distribution and dislocation density, which is convenient for analyzing the details of dislocation initiation and expansion.

**Figure 5:**
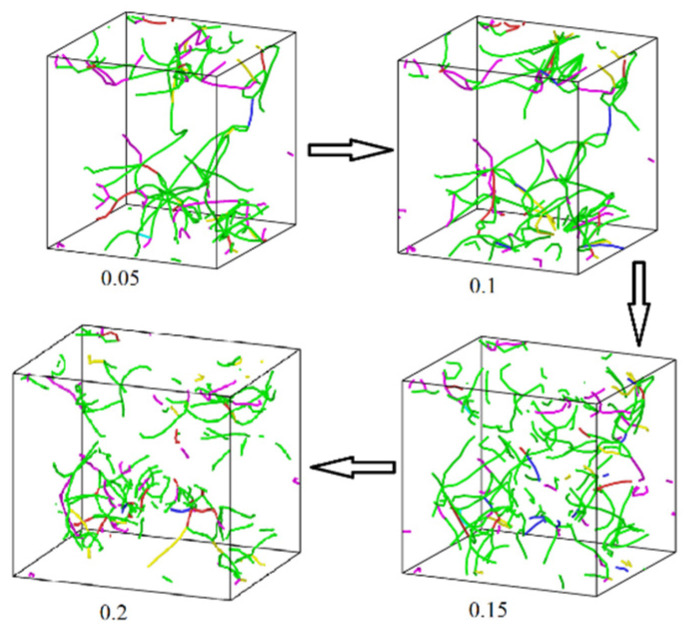
The distribution of the dislocation lines in sintered pure Cu NP solder paste under different tensile strains. The different colored lines represent the dislocations with different Burgers vectors, where green is a Shockley, pink is a stair-rod, light blue is a Frank, yellow is a Hirth, dark blue is a perfect dislocation, and red is other.

**Figure 6:**
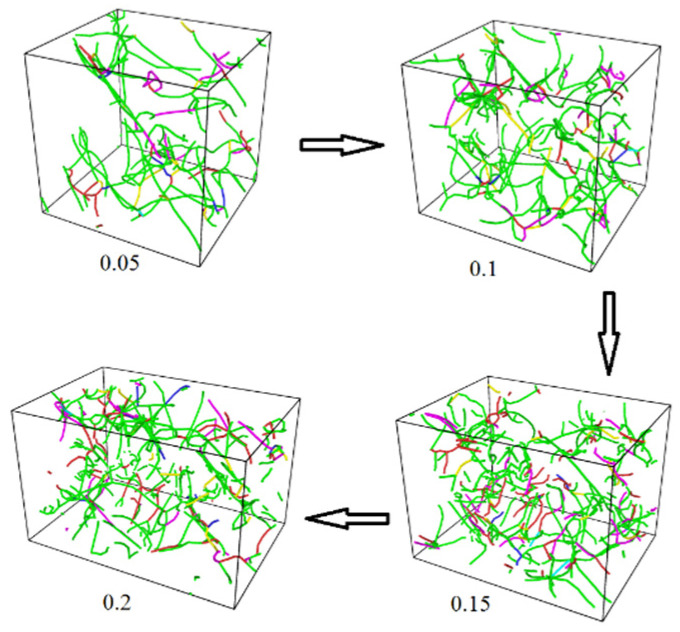
The distribution of the dislocation lines in sintered Cu NP/GNP composite solder paste under different tensile strains. The different colored lines represent the dislocations with different Burgers vectors, where green is a Shockley, pink is a stair-rod, light blue is a Frank, yellow is a Hirth, dark blue is a perfect dislocation, and red is other.

**Figure 7:**
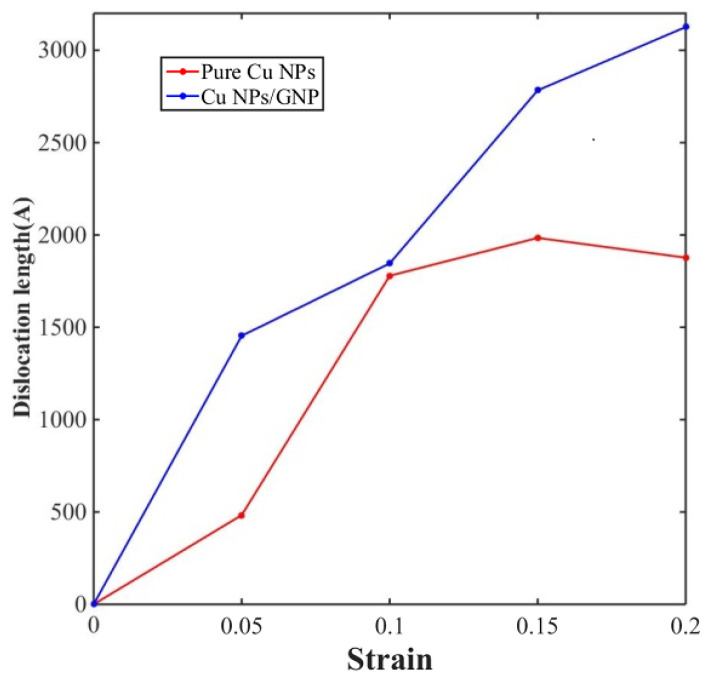
The dislocation length of the sintered pure Cu NP solder paste and Cu NP/GNP composite solder paste at different tensile strains.

Figure 5 and Figure 6 display the distribution of the dislocation lines of pure Cu NP solder paste and Cu NP/GNP composite solder paste during the tensile process under the application of different tensile strains of 0.05, 0.1, 0.15, and 0.2. Additionally, Figure 7 shows that at these different strain levels, the total lengths of the dislocation lines in the sintered pure Cu NP solder paste were 483 Å, 1779 Å, 1984 Å, and 1877 Å, respectively. The total lengths of the dislocation lines in the sintered Cu NP/GNP composite solder paste were 1454 Å, 1846 Å, 2783 Å, and 3126 Å, respectively. Compared with the Cu NP/GNP composite solder paste, the total lengths of the dislocation lines in the pure Cu NP solder paste were shorter during the stretching process. By adding GNP to the pure Cu NP solder paste, the total length and dislocation density of the dislocation lines can be increased. Since the addition of GNP can increase the dislocation density of the composite solder paste sinter, it plays an important role in preventing dislocation movement. Consequently, this effect results in higher compressive strength of the Cu NP solder paste with GNP.

### 3.2. The Impact of the Different Sintering Parameters on the Mechanical Properties

#### 3.2.1. Addition of GNP

**Figure 8:**
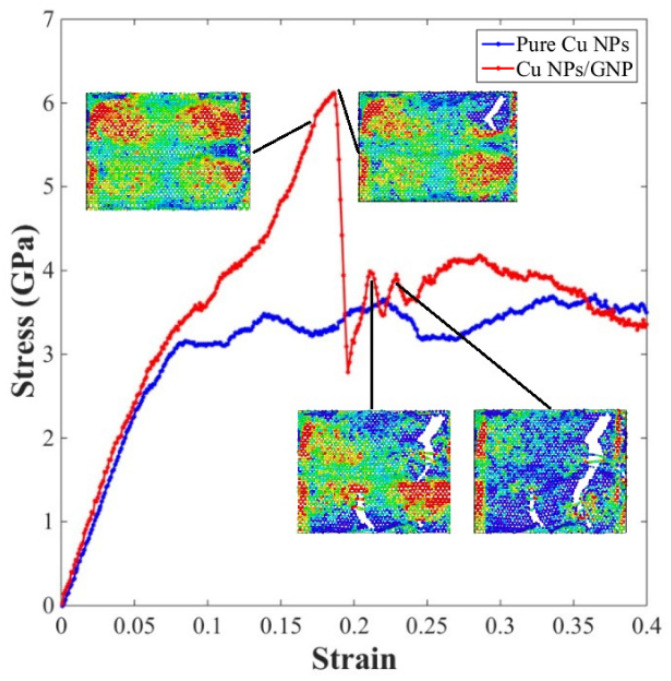
Uniaxial tensile stress–strain diagram of sintered structures with different sintering models.

The stress–strain response of the sintered pure Cu NP solder paste and the Cu NP/GNP composite solder paste was obtained by applying uniaxial tensile deformation to the sintered structures. The stress–strain curves of these two different sintering models are illustrated in Figure 8. In this work, the yield strength of the sintered structures was obtained based on the initial yield strength, which can be determined as the first peak observed in the stress–strain diagram. The yield strength values of the sintered pure Cu NP solder paste and the Cu NP/GNP composite solder paste were 3.65 GPa and 6.12 GPa, respectively. Compared with the pure Cu NP solder paste sintered structures, the yield strength of the composite solder paste sintered structures with GNP added increased by 67.7%.

**Figure 9:**
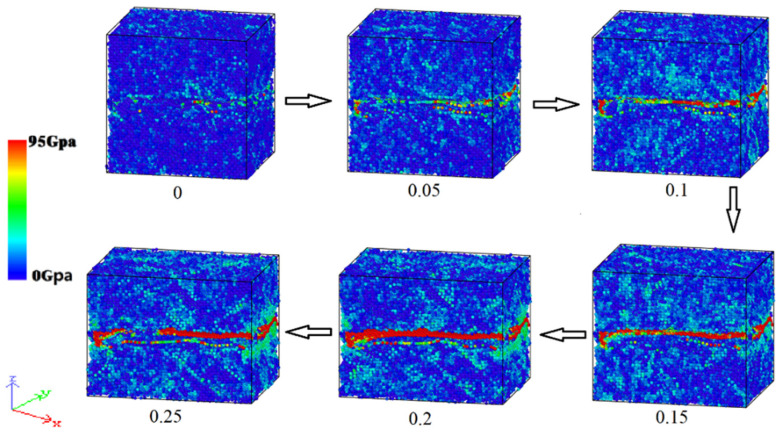
Atomic stress diagram of sintered Cu NP/GNP composite solder paste in uniaxial tensile process along with *X*-axis.

**Figure 10:**
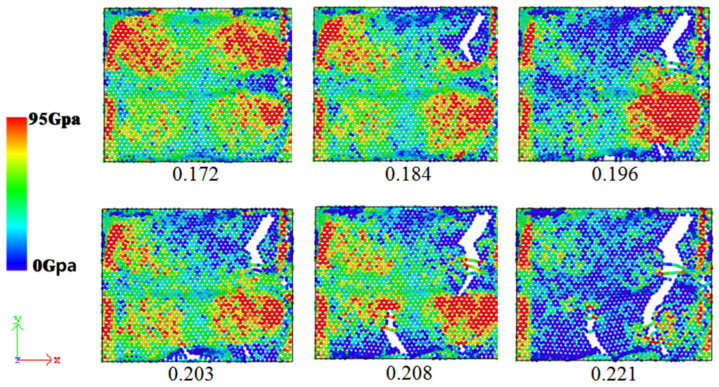
Atomic stress of GNP during uniaxial tension along with *X*-axis under different tensile strains.

To further investigate the impact of graphene nanoplatelet addition on the mechanical properties of sintered Cu NP/GNP composite solder paste, the atomic stress in the tensile process of the sintered solder paste was calculated and verified by using the OVITO (V2.9.0) software package. The atomic stress diagram is shown in Figure 9. According to the atomic stress diagram, it can be observed that the stress of the sintered solder paste during the tensile process is mainly concentrated on the graphene nanoplatelet, which bears more tensile stress. To better observe the deformation and fracture process of the graphene nanoplatelet in the tensile process, the stress state of the graphene nanoplatelet was considered separately from the top view, as depicted in Figure 10. When the strain was 0.184, the graphene nanoplatelet started to break, and the stress of the sintered Cu NP/GNP composite solder paste started to drop sharply. When the strain reached 0.2, the graphene nanoplatelet gradually cracked from multiple edges, which corresponds to the multiple wave breaks in the stress–strain diagram.

#### 3.2.2. Sintering Temperature

**Figure 11:**
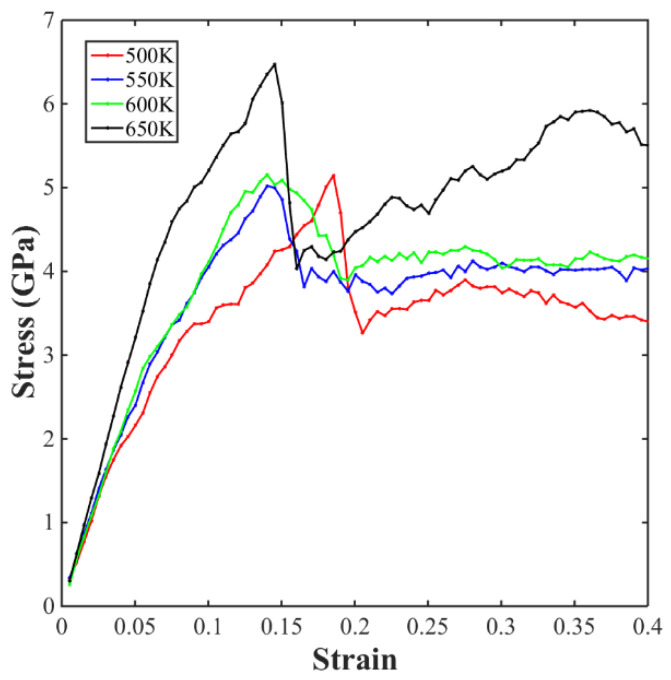
Uniaxial tensile stress–strain curve of the sintered paste at different sintering temperatures.

In this work, the yield strength of sintered structures was estimated based on the initial yield strength, which can be determined as the first peak observed in the stress–strain plot. Figure 11 shows a stress–strain curve of the Cu NP/GNP composite solder paste sintered at different sintering temperatures. As can be observed, Young’s modulus increased with increasing temperature, which means that the sintered solder paste has stronger elastic properties at higher temperatures. It can also be observed that the yield strength of the sintered solder paste increased with increasing temperature, and the sample sintered at a 650 K sintering temperature reached the highest yield strength. It can be seen that the yield strength of the sintering temperature between 500 K and 600 K had no obvious change. When the temperature was increased from 600 K to 650 K, the yield strength increased by nearly 30%.

#### 3.2.3. Tensile Strain Rate

**Figure 12:**
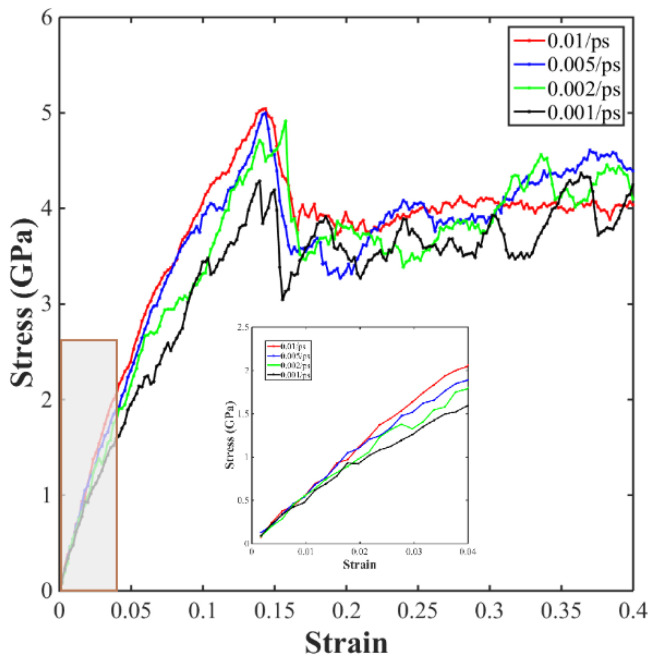
Uniaxial tensile stress–strain curves of the sintered composite solder paste with different tensile strain rates.

To investigate the relationship between the mechanical properties and the tensile strain rate, the stress and strain of the sintered solder paste were investigated under different tensile strain rates. As can be ascertained from Figure 12, the tensile strength and fracture strain of the sintered composite solder paste increased with an increasing tensile strain rate. In addition, the time taken to reach the ultimate tensile strength decreased with an increase in the tensile strain rate, and the Young’s modulus of the sintered solder paste decreased slowly with an increase in the strain rate. By analyzing the tensile results of the Cu NP/GNP sintered composite solder paste under different strain rates, it was found that the tensile strength of the sintered composite solder paste gradually increased with an increase in the strain rate, but the initial equivalent modulus remained basically unchanged.

## 4. Conclusions

In this paper, the molecular dynamics simulation method was used to study the sintering mechanism of Cu NP/GNP composite solder paste. By applying uniaxial tensile simulation to the sintered structures and studying its stress–strain curve, the influence of the different sintering parameters on the mechanical properties of the composite solder paste sintered structures was systematically examined. The main conclusions are summarized below:

(1) The addition of GNP and sintering temperature has a great influence on the mechanical properties of the composite paste. Compared with the pure Cu NP solder paste sintered structures, the yield strength of the composite solder paste sintered structures with GNP added increased by 67.7%. The yield strength of the composite solder paste sintered body increased with an increase in temperature, reaching the highest yield strength at a sintering temperature of 650 K.
(2) The tensile strain rate has a certain influence on the tensile process of Cu NP/GNP composite solder paste. A higher strain rate leads to a higher tensile strength and fracture strain of the composite.
(3) Compared with the process of holding temperature and pressure, the influence of the heating and cooling rates in the ascending or descending process on atomic diffusion is not significant. To improve the atomic diffusion, it is necessary to properly increase the holding time and sintering temperature, and reduce the duration of the temperature and pressure increase and decrease.
(4) GNP can effectively prevent dislocation movement and achieve a good strengthening effect. Therefore, the strengthening mechanism of Cu NP/GNP composite solder paste can be attributed to the dislocation strengthening mechanism.

## Figures and Tables

**Table 1:** Initial size and number of atoms of simulation model.

Material	Size (Å)	Number of Atoms
Cu NPS	25 (radius)	5556
GNP	90 × 90	3402

**Table 2:** Sintering and tension process parameters.

Simulation Process	Time (ps)	Temperature (K)	Temperature Change Rate (K/ps)	Pressure (MPa)	Pressure Change Rate (MPa/ps)	Ensemble
		Start End		Start End		
① Relaxation	50	300 300	0	0.1 0.1	0	NPT
② T and P rising	300	300 500	0.67	0.1 600	2	NPT
③ T and P holding	300	500 500	0	600 600	0	NPT
④ T and P reducing	300	500 300	0.67	600 0.1	2	NPT
⑤ T and P holding	300	300 300	0	0.1 0.1	0	NPT
⑥ Quasi-static tensile	40	300 300	0	0.1 0.1	0	NPT

## Data Availability

The data and code used in this paper are available from the corresponding author upon reasonable request.
